# EBV BART MicroRNAs Target Multiple Pro-apoptotic Cellular Genes to Promote Epithelial Cell Survival

**DOI:** 10.1371/journal.ppat.1004979

**Published:** 2015-06-12

**Authors:** Dong Kang, Rebecca L. Skalsky, Bryan R. Cullen

**Affiliations:** 1 Department of Molecular Genetics and Microbiology, Duke University Medical Center, Durham, North Carolina, United States of America; 2 Center for Virology, Duke University Medical Center, Durham, North Carolina, United States of America; University of North Carolina at Chapel Hill, UNITED STATES

## Abstract

Epstein-Barr virus (EBV) is a ubiquitous human γ-herpesvirus that can give rise to cancers of both B-cell and epithelial cell origin. In EBV-induced cancers of epithelial origin, including nasopharyngeal carcinomas (NPCs) and gastric carcinomas, the latent EBV genome expresses very high levels of a cluster of 22 viral pre-miRNAs, called the miR-BARTs, and these have previously been shown to confer a degree of resistance to pro-apoptotic drugs. Here, we present an analysis of the ability of individual miR-BART pre-miRNAs to confer an anti-apoptotic phenotype and report that five of the 22 miR-BARTs demonstrate this ability. We next used photoactivatable ribonucleoside-enhanced crosslinking and immunoprecipitation (PAR-CLIP) to globally identify the mRNA targets bound by these miR-BARTs in latently infected epithelial cells. This led to the identification of ten mRNAs encoding pro-apoptotic mRNA targets, all of which could be confirmed as valid targets for the five anti-apoptotic miR-BARTs by indicator assays and by demonstrating that ectopic expression of physiological levels of the relevant miR-BART in the epithelial cell line AGS resulted in a significant repression of the target mRNA as well as the encoded protein product. Using RNA interference, we further demonstrated that knockdown of at least seven of these cellular miR-BART target transcripts phenocopies the anti-apoptotic activity seen upon expression of the relevant EBV miR-BART miRNA. Together, these observations validate previously published reports arguing that the miR-BARTs can exert an anti-apoptotic effect in EBV-infected epithelial cells and provide a mechanistic explanation for this activity. Moreover, these results identify and validate a substantial number of novel mRNA targets for the anti-apoptotic miR-BARTs.

## Author Summary

One important innate immune response to viral infection is apoptosis, also called programmed cell death, whereby the infected cells commit suicide rather than serve as factories for virus production. As a result, many viruses have developed strategies to inhibit apoptosis. Here, we demonstrate that five of the Epstein-Barr virus (EBV) miR-BART microRNAs that are expressed in EBV-transformed epithelial cell tumors display anti-apoptotic activity. We have identified ten cellular mRNAs that are bound and downregulated by one of these five anti-apoptotic microRNAs and show that this downregulation can explain the observed reduction in apoptosis in miR-BART-expressing cells. Together, these data demonstrate that the EBV miR-BARTs can help sustain latently EBV-infected cells in the face of pro-apoptotic innate immune signals and this may explain the resistance to DNA damaging agents, including chemotherapeutics and radiation, seen in a subset of EBV-induced epithelial tumors.

## Introduction

MicroRNAs (miRNAs) are 22 ± 2 nucleotide (nt) non-coding RNAs that are expressed by all multicellular eukaryotes as well as by several viruses [1–3]. MiRNAs are generally initially transcribed by RNA polymerase II in the form of a long primary miRNA (pri-miRNA) precursor that is sequentially processed by the RNase III enzymes Drosha, in the nucleus, to generate the pre-miRNA intermediate and Dicer, in the cytoplasm, to yield the mature miRNA [1, 4]. Upon loading into the RNA-induced silencing complex (RISC), the miRNA serves as a guide RNA to direct RISC to partially complementary target sites [5]. Particularly important in this regard is the miRNA seed sequence, extending from position 2 to 8 on the miRNA, which is exposed during mRNA binding by RISC and plays a key role in target mRNA recognition [5, 6]. Because seed sequence complementarity to an mRNA target is generally not only necessary but frequently also sufficient for effective RISC recruitment, it is predicted that each miRNA functionally interacts with >100 mRNA targets. RISC binding in turn results in the translational inhibition and partial destabilization of the target mRNA [5]. The accurate identification of these mRNA targets, and more importantly, the discovery of mRNA targets that are phenotypically relevant, remains the most difficult challenge in understanding miRNA function. This is particularly difficult in the case of virally encoded miRNAs as these are subject to rapid evolution and, unlike cellular miRNA target sites, which have co-evolved with host cell miRNAs, cellular mRNA targets for viral miRNAs are generally not evolutionarily conserved. Efforts to identify important mRNA targets for viral miRNAs have therefore generally followed one of two approaches, which have been respectively referred to as the “bottom up” and “top down” approach [2]. In the “top down” approaches, the investigator first identifies a phenotype exerted by a miRNA then seeks to determine which mRNA target(s) is responsible for this phenotype. Conversely, in the “bottom up” approach, the investigator first uses computational methods or experimental techniques, such as microarray analysis or a cross-linking/immunoprecipitation approach, to globally identify mRNA targets for a given viral miRNA then seeks to confirm that the phenotypic effect predicted upon downregulation of a given mRNA target is actually observed. These approaches are not, of course, mutually exclusive as tools for the global identification of mRNA targets for a given viral miRNA can provide critical information for efforts to identify the mRNA target(s) that explain a miRNA phenotype.

Epstein-Barr virus (EBV) encodes two miRNA clusters that are differentially expressed during latent EBV infection [7–10]. In latency III, as seen for example in lymphoblastoid cell lines (LCLs) of primary B-cell origin, EBV expresses a high level of the three viral pre-miRNAs encoded in the miR-BHRF1 cluster and moderate levels of the 22 pre-miRNAs encoded in the miR-BART cluster [7, 10, 11]. Consistent with this expression pattern, mutational inactivation of the miR-BHRF1 cluster severely impairs B-cell transformation by EBV, with the resultant LCLs showing a slow growth phenotype, while loss of all 22 miR-BARTs has at most a modest effect on B-cell transformation [12–15]. Conversely, in EBV-transformed epithelial cells that are in latency II, including nasopharyngeal carcinoma (NPC) cells and EBV-induced gastric carcinomas, the miR-BHRF1 cluster is not expressed while the miR-BARTs are transcribed at substantial levels [7, 9, 10, 16, 17]. Whether the miR-BART miRNAs are required for the transformation of primary human epithelial cells by EBV remains unclear, due to the lack of good *in vitro* systems to study this process. However, analysis using the gastric carcinoma cell line AGS strongly suggests that this is likely to be the case. AGS cells are normally EBV-negative but can be readily infected by EBV to establish a latent infection marked by high level expression of the miR-BARTs, as well as the viral EBNA1 protein and the EBER non-coding RNAs, but only very low levels of the other viral latent proteins, including LMP1 and EBNA2 [18, 19]. Strikingly, EBV+ AGS cells show enhanced anchorage independent cell growth and the ectopic expression of the miR-BART miRNAs in AGS cells also inhibits apoptosis [18, 20, 21]. This latter result is consistent with a number of reports that have provided evidence for the downregulation of pro-apoptotic cellular genes by individual miR-BART miRNAs [14, 20–24]. However, at present a full understanding of how the EBV miR-BART miRNAs inhibit apoptosis to promote the viability of EBV-infected epithelial cells remains elusive. Here, we report a systematic effort to identify pro-apoptotic mRNA targets for the EBV miR-BART miRNAs. We demonstrate that at least five of the 22 miR-BART pre-miRNAs have anti-apoptotic activity and we identify seven pro-apoptotic cellular mRNA targets, six of them novel, that contribute significantly to the observed anti-apoptotic phenotype. Together, these data represent a substantial increase in our understanding of the role of the miR-BART miRNAs in promoting EBV infection and latency.

## Results

Although no system for the study of transformation of primary human epithelial cells by EBV is currently available, the human EBV-negative epithelial cell line AGS, derived from a gastric carcinoma, has emerged as a useful model system [18]. In particular, infection of AGS with EBV, which results in latently EBV infected AGS cells that express high levels of the miR-BART miRNAs, has been associated with enhanced anchorage independent growth *in vitro*, enhanced tumor formation *in vivo* in mice and a reduction in apoptosis [18, 21, 25]. As apoptosis is well established as a key innate immune response to viral infection [26, 27], and given several reports suggesting that individual miR-BARTs can target specific pro-apoptotic cellular genes to promote cell survival [14, 20–24], we decided to systematically analyze the anti-apoptotic potential of the 22 miR-BART pre-miRNAs in the human epithelial cell line AGS.

**Fig 1:**
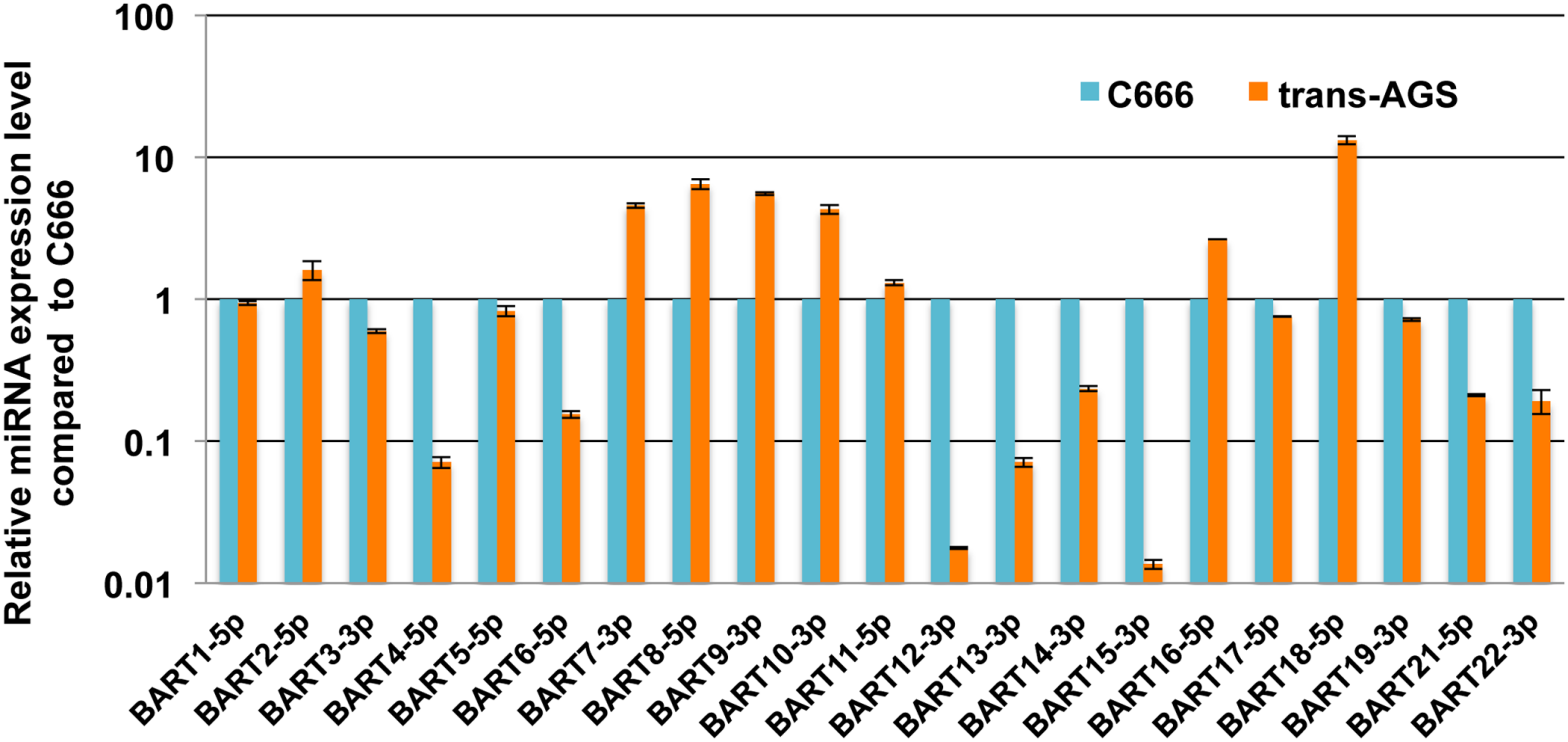
Expression of individual EBV miR-BARTs in human AGS cells. AGS cells were transduced with lentiviral vectors designed to express individual miR-BART miRNAs. After selection for antibiotic resistance, cells were sorted for high turboRFP expression and expanded. miR-BART expression levels were then quantified by qRT-PCR and are given here in comparison to the level detected in the EBV-infected NPC cell line C666, which was set at 1. Average of 2 replicates with SD indicated.

The phenotypic effect of a given miRNA is in part determined by the expression level of the miRNA relative to its potential pool of mRNA targets so that ectopic overexpression of a given miRNA can give rise to phenotypic effects that are not seen at physiological levels of expression [28]. Therefore, to identify EBV miR-BART miRNAs with anti-apoptotic potential, we decided to express each of the 22 miR-BARTs at approximately physiological levels using lentiviral miRNA expression vectors, constructed as previously reported by inserting the entire pri-miR stem-loop, together with ~100 bp of 5’ and 3’ flanking sequence, into the 3’ UTR of the turbo red fluorescent protein (turboRFP) gene present in pTRIPZ [29, 30]. After selection for the included puromycin marker, the cells were sorted for high turboRFP expression (upper 30%) and expanded. To analyze the expression level of each miR-BART miRNA in each AGS transductant, we harvested total RNA from each culture and then used qRT-PCR to compare the level of expression to that seen in the EBV latency II NPC cell line C666. As may be observed in Fig 1, we achieved stable expression of levels of several of the miR-BARTs in AGS cells that were comparable to the endogenous levels seen in C666. Eight of the miR-BARTs (miR-BART 2, 7, 8, 9, 10, 11, 16 and 18) were expressed at levels slightly above that seen in C666 while a further nine (miR-BART1, 3, 5, 6, 14, 17, 19, 21 and 22) were expressed at levels close to, but slightly below, the levels seen in C666. Finally, the remaining five miR-BARTs were either expressed at levels >10-fold lower than seen in C666 (miR-BART4, 12, 13 and 15) or were not detected (miR-BART20). Therefore, these latter transductants can be viewed essentially as negative controls.

**Fig 2:**
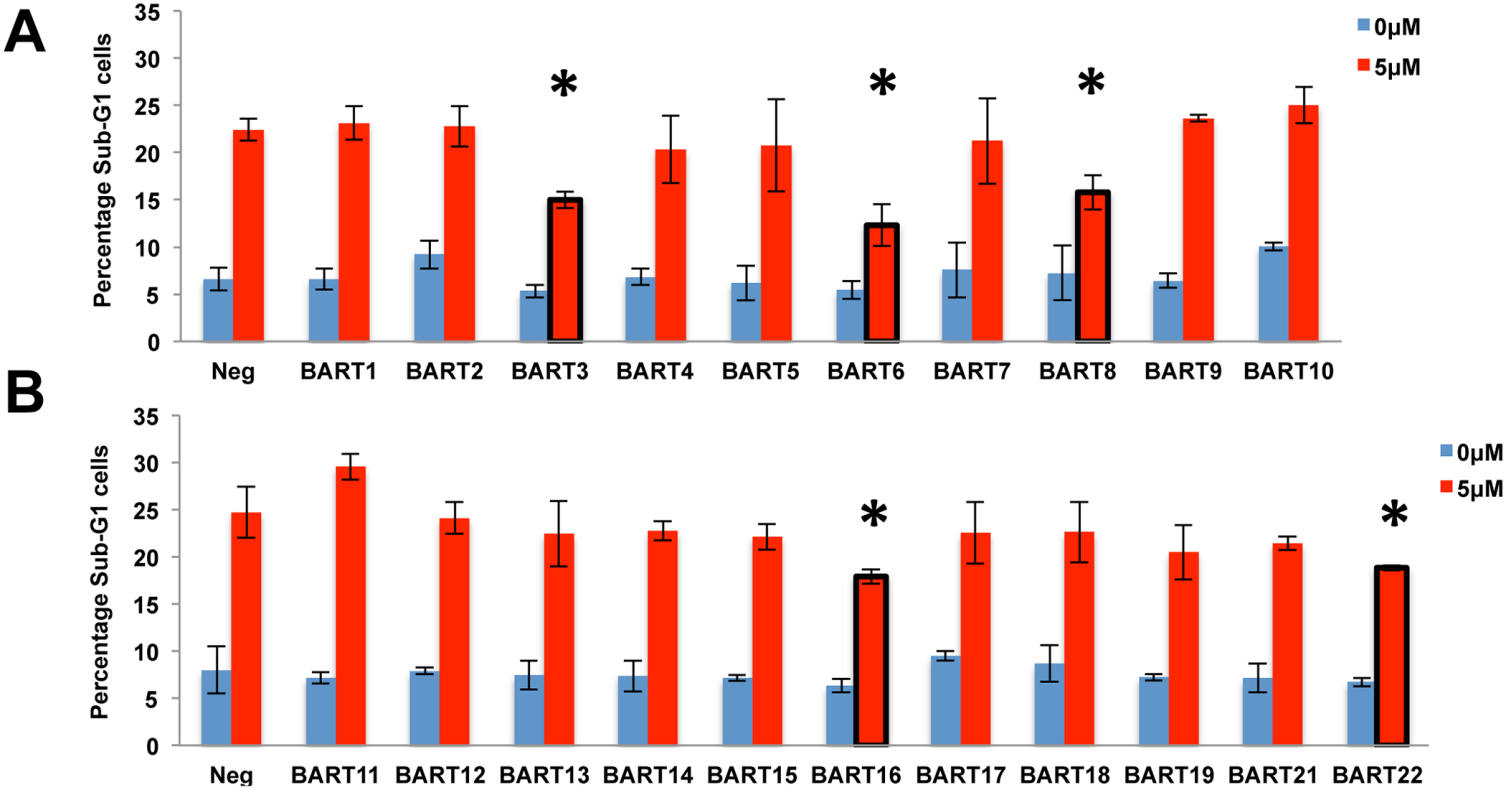
Several miR-BART miRNAs exert an anti-apoptotic phenotype. (A) Percentage representation of the Sub-G1 population in AGS cell cultures individually expressing miR-BART1 to BART10 either without drug treatment or after treatment for 24 h with 5 μM etoposide. Neg: negative control lentiviral vector. Average of 3 experiments with SD indicated. (*: Students’ T-Test, p<0.05). (B) Similar to panel A except looking at AGS cells engineered to express miR-BART 11 to 19 or miR-BART 21 or 22.

Previously, Marquitz et al. [20] reported that ectopic expression of clusters of EBV BART miRNAs in AGS cells (either miR-BART1, 3, 4, 5, 6, 15, 16 and 17 or miR-BART7, 8, 9, 10, 11, 12, 13, 14, 18, 19 and 20) confers resistance to apoptosis induced by treatment with etoposide and we therefore initially examined whether expression of any of these individual BART miRNAs would exert a similar phenotypic effect. As shown in Fig 2A and 2B, we observed a significant reduction in the level of apoptotic cells in the AGS cultures expressing pre-miR-BART3, 6, 8, 16 and 22 when compared to the other 18 cultures. This result, which was initially obtained by quantitation of the sub-G1 population of AGS cells by FACS, could also be largely confirmed by Western blot analysis for cleaved and uncleaved PARP expression, with the AGS cultures expressing miR-BART6, 8, 16 or 22 showing significantly reduced levels of cleaved PARP after etoposide treatment (we did not see a statistically significant reduction in the case of miR-BART3) (S1A and S1B Fig).

The inhibition of etoposide-induced apoptosis observed in Fig 2 and S1 Fig in the cultures expressing miR-BART6, 8, 16, 22, and possibly also miR-BART3, presumably reflects the downregulation of one or more mRNAs with pro-apoptotic potential by each of these viral miRNAs. A number of possible mRNA targets for individual miR-BARTs have been reported, some of which have clear pro-apoptotic potential [14, 20–24]. We therefore wondered if the anti-apoptotic activity of the five miR-BART miRNAs defined in Fig 2 could be explained by these previously published target mRNAs. To address this question, we generated indicator constructs in which the 3’ UTR of a cellular gene of interest (either the complete 3’UTR or a segment of 540 bp or more, see S1 Table) was inserted 3’ to the firefly luciferase (FLuc) indicator gene [29]. Then, 293T cells were co-transfected with the relevant indicator construct, a miR-BART miRNA or control expression vector and an internal control plasmid expressing Renilla luciferase (RLuc). At ~72 h post-transfection, the cells were lysed and the relative expression of FLuc and RLuc, in the presence and absence of the miR-BART miRNA, quantified. In general, our experience has been that this assay format produces a readily detectable, >20% reduction in FLuc expression that is both reproducible and statistically significant. All miR-BART miRNA expression vectors tested were fully biologically active, as previously demonstrated [30] by their ability to downregulate a similar FLuc-based indicator construct containing a perfectly complementary target site inserted into the 3’UTR.

**Fig 3:**
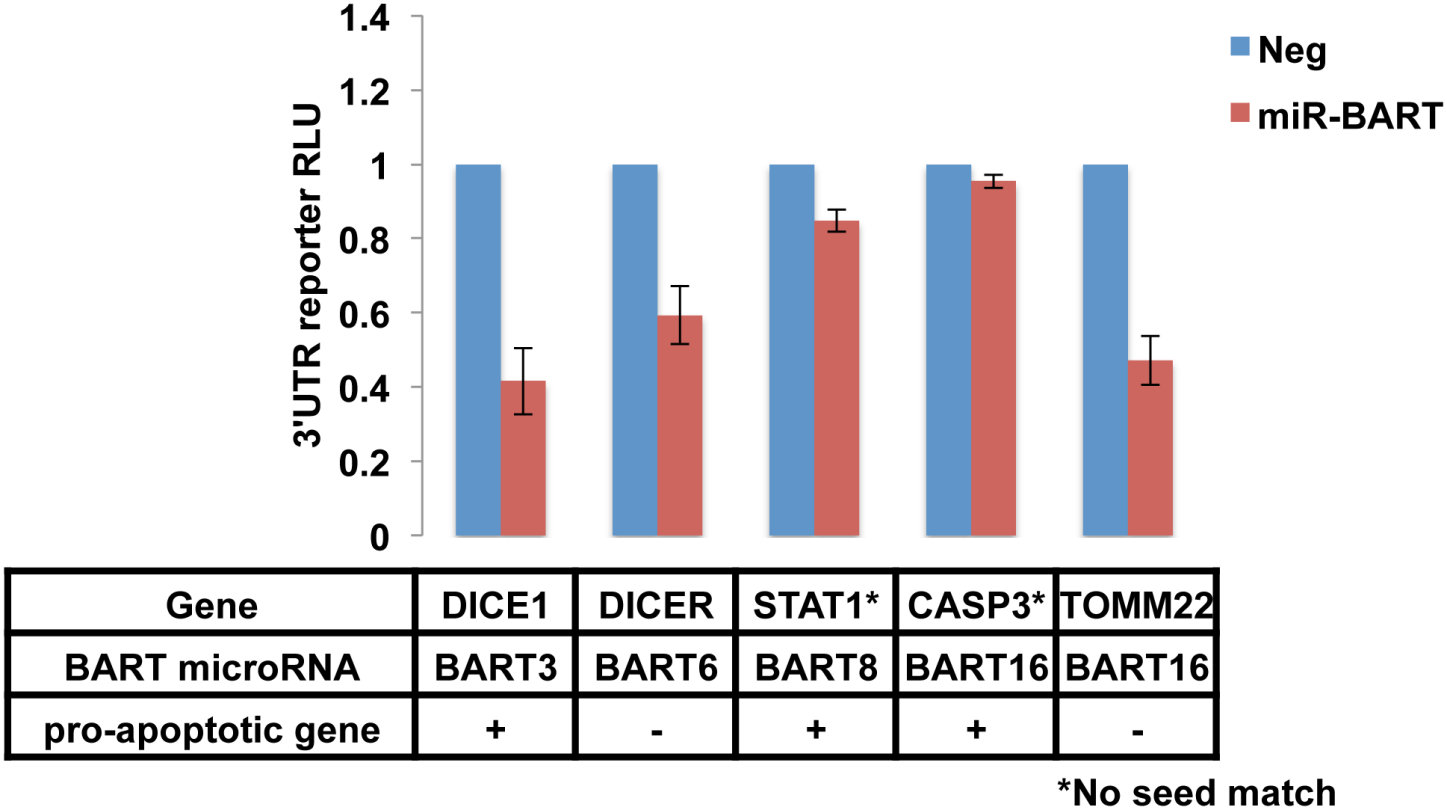
Published targets of miR-BARTs do not fully explain an anti-apoptotic phenotype. Upper panel: FLuc-based reporter plasmids containing the 3’UTR of the indicated cellular mRNAs were co-transfected into 293T cells along with a vector expressing the indicated miR-BART, or an empty vector, as well as an RLuc-based internal control plasmid. At 72 h post-transfection, induced Fluc and RLuc activities were determined and normalized to RLuc. The negative control was then set at 1. Average of three independent experiments with SD indicated. Lower panel: five published targets of anti-apoptotic miR-BARTs with predicted pro-apoptotic activity indicated by +. (* Indicates the absence of a complete seed match between the 3’UTR and the miR-BART indicated).

As shown in Fig 3, the 3’UTRs of the cellular mRNAs encoding DICE1, a proposed target for miR-BART3 [23], Dicer, a proposed target for miR-BART6 [31], and TOMM22, a proposed target for miR-BART16 [32], all produced a readily detectable inhibitory effect on FLuc expression when present in *cis* in cells expressing the cognate miR-BART miRNA. In contrast, we did not see a significant reduction mediated by the 3’UTR of STAT1, a proposed target for miR-BART8 [33], or by the 3’UTR of caspase 3 (CASP3), a proposed target for miR-BART16 [14]. We note that neither of these 3’UTRs contains an intact seed target for miR-BART8 or miR-BART16, respectively, so the observed lack of inhibition was not unexpected. While the 3’ UTR of CASP3 has been suggested to function as a target for several miRNAs encoded by the human γ-herpesvirus KSHV [34], we did not observe a significant inhibition of an FLuc indicator bearing the 3’UTR of CASP3 in the presence of miR-BART3, 6, 8, 16 or 22, although the modest, ~20% repression seen with miR-BART3 did approach significance (S2B Fig). Similarly, the 3’UTR of the pro-apoptotic cellular gene BIM, another proposed miR-BART target [20], also did not function as an effective target for miR-BART3, 6, 8, 16 or 22 in this indicator assay (S2A Fig). It remains possible that the simultaneous expression of several miR-BARTs might induce a more marked inhibitory effect. Indeed, Marquitz et al. [20] reported that an analogous indicator containing the BIM 3’UTR was not affected by co-expression of any individual miR-BART miRNA but was inhibited by the simultaneous expression of multiple BART miRNAs. However, as the anti-apoptotic phenotypes shown in Fig 2 result from the expression of individual miR-BART miRNAs, it is apparent that mRNA targets relevant to these phenotypes must be significantly responsive to these individual miRNAs. Based on these results, it therefore appears that while the anti-apoptotic effect observed for miR-BART3 (Fig 2) might be explained by downregulation of the pro-apoptotic gene product encoded by cellular DICE1 [23], the analogous effects exerted by miR-BART6, miR-BART8, miR-BART16 or miR-BART22 are not readily accounted for by previously reported mRNA targets for those miRNAs. Specifically, while we could confirm downregulation of Dicer mRNA function by miR-BART6 and TOMM22 mRNA function by miR-BART16 (Fig 3), neither of these two proteins is known to be pro-apoptotic [31, 35]. Conversely, we did not observe significant downregulation mediated by the 3’UTRs of the pro-apoptotic genes STAT1, CASP3 or BIM in the presence of any of these individual miRNAs (Fig 3 and S2 Fig). We therefore next sought to globally identify the mRNA targets for the EBV miR-BART miRNAs in the naturally EBV-infected epithelial cell line C666 using the previously described photoactivatable ribonucleoside-enhanced crosslinking and immunoprecipitation (PAR-CLIP) technique [11, 36, 37].

### Identification of novel pro-apoptotic cellular mRNA targets for the anti-apoptotic miR-BART miRNAs

**Fig 4:**
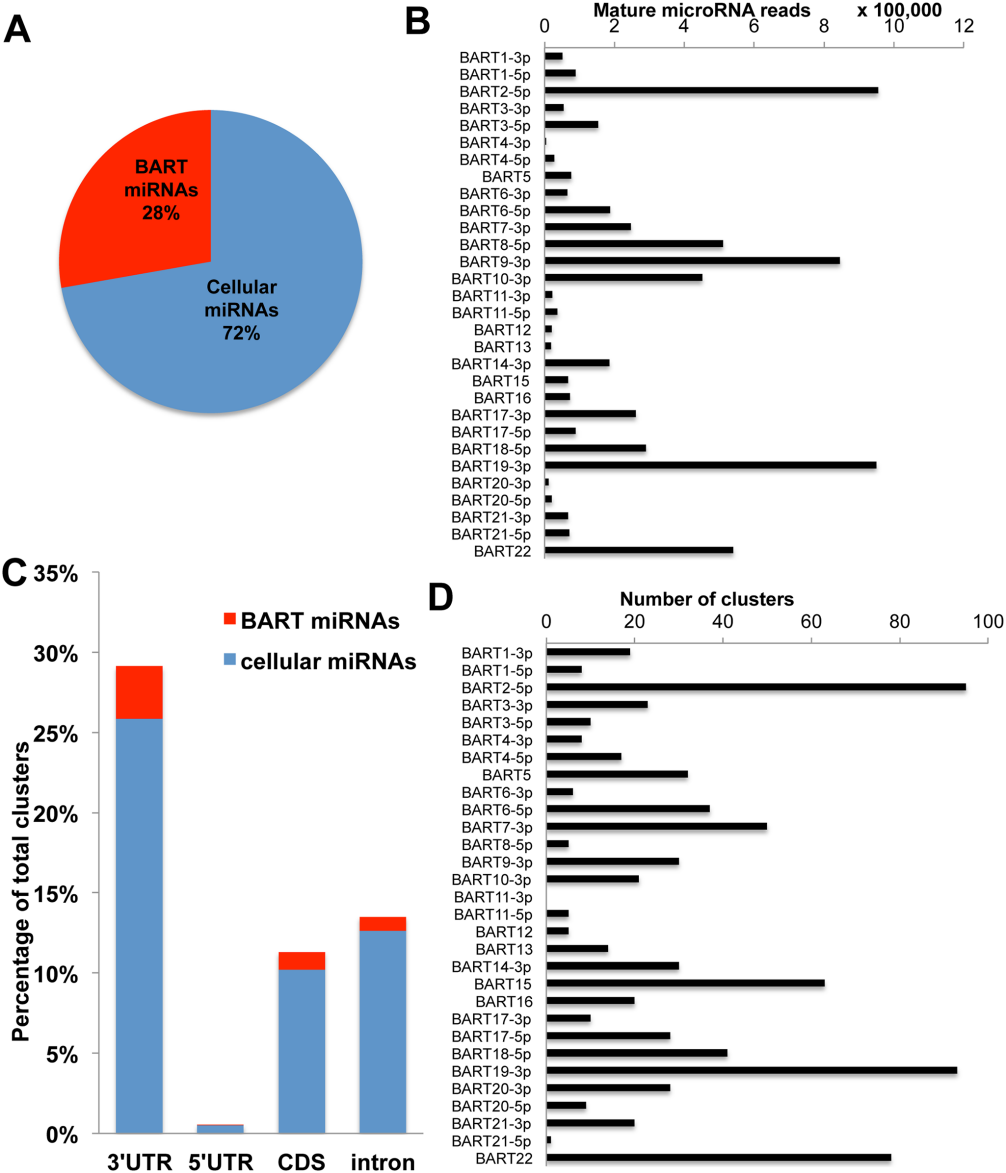
PAR-CLIP analysis of the NPC cell line C666 identifies 792 putative miR-BART 3’UTR interaction sites. (A) Deep sequencing of small (~18 to 24 nt long) RNAs expressed in C666 cells identified ~23.9 X 10^6^ miRNA reads of which 28% mapped to the EBV BART locus. (B) Assignment of deep sequencing reads to individual miR-BART miRNAs or highly expressed miR-BART passenger strands. (C) Distribution of miRNA binding clusters for cellular and EBV miR-BART miRNAs identified by PAR-CLIP in terms of their genomic assignment. (D) Assignment of miRNA binding clusters to individual miR-BARTs or miR-BART passenger strands.

We initially performed deep sequencing of the small RNA population (~18 to ~24 nt in size) in C666 cells, as previously described [11, 37]. This resulted in a total of 26.6x10^6^ reads, of which 25.8x10^6^ (~97%) mapped to either the human or EBV genome (See S2 Table for the complete data set). Of these, 23.9x10^6^ (~93%) represent known mature miRNAs or miRNA passenger strands, with 6.7x10^6^ (~28%) mapping to the EBV miR-BART locus and the remaining 17.2 x10^6^ reads (~72%) representing known human miRNAs (Fig 4A). Among the miR-BARTs, we recovered reads from all 22 known miR-BART miRNA and miRNA passenger strands, but only those miRNA passenger strands representing ≥10% of the total reads derived from a given pre-miR-BART intermediate are shown in Fig 4B. The most highly expressed miR-BARTs detected in C666 cells were miR-BART2-5p, miR-BART9-3p and miR-BART19-3p.

**Fig 5:**
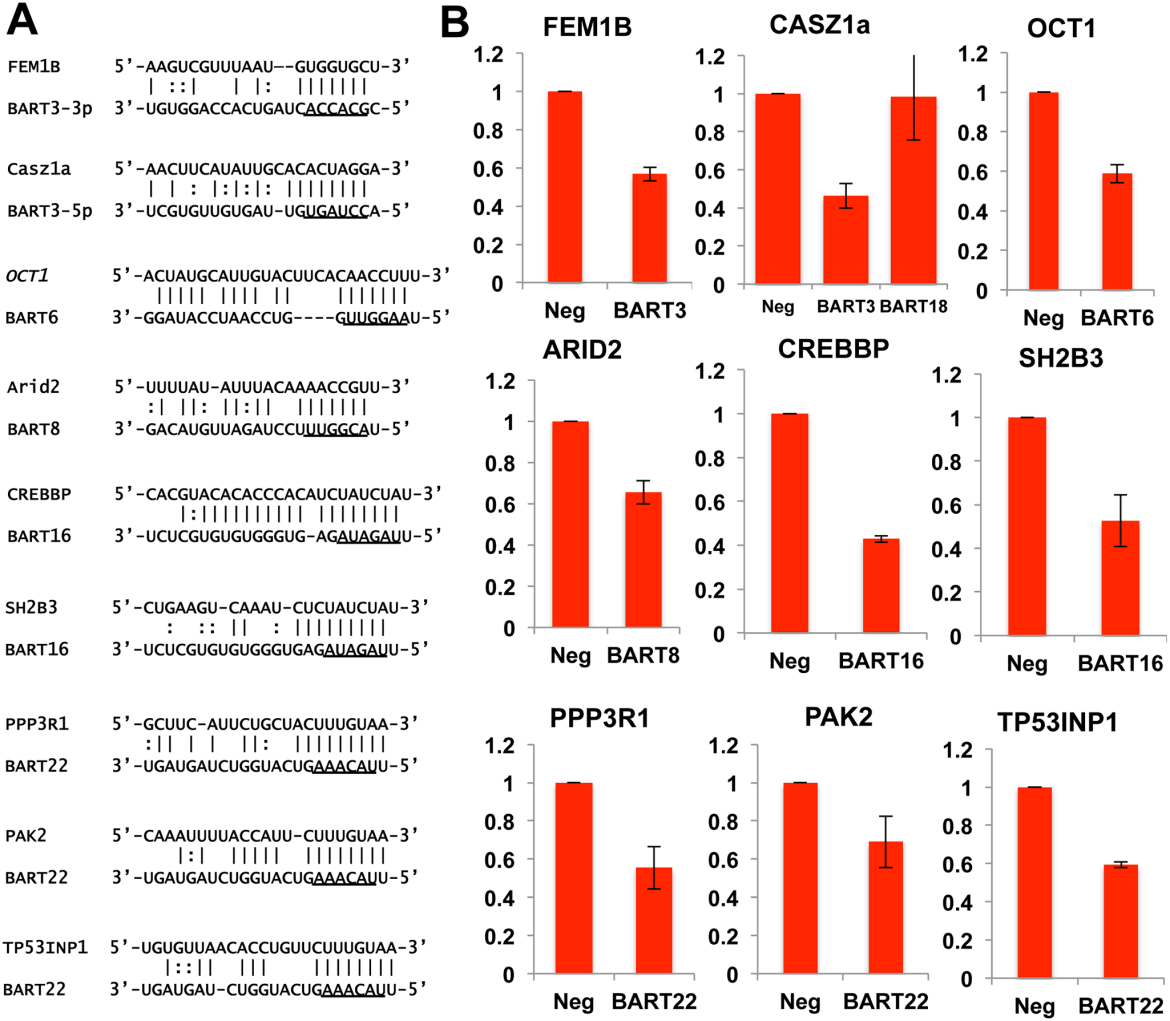
PAR-CLIP analysis of RISC binding sites in C666 cells identifies potentially pro-apoptotic mRNAs targeted by anti-apoptotic miR-BARTs. (A) Alignment of 3’UTR Ago binding clusters of nine putatively pro-apoptotic human genes to the 5 anti-apoptotic miR-BARTs. Underlined sequences represent the 7-nt seed regions of the miR-BARTs, which in all cases are fully complimentary to the target. (B) FLuc-based reporter plasmids containing the indicated 3’UTRs were repressed upon co-expression of the indicated anti-apoptotic miR-BART pre-miRNA. A predicted site for miR-BART18, which is not anti-apoptotic, in the 3’UTR of CASZ1a proved to be inactive. Average of 3 experiments with SD indicated.

Next we performed PAR-CLIP to globally identify the mRNA targets for the miR-BARTs in C666 cells using an antibody that immunoprecipitates all four human Argonaut (Ago) proteins. The PAR-CLIP library gave 16.7x10^6^ reads, of which 6.6x10^6^ could be mapped to a unique sequence present in either the human or EBV genome (see S3 Table for the complete data set). Computational definition of binding site clusters and assignment to expressed miRNAs [38] revealed that the majority of both the cellular miRNA and miR-BART clusters mapped to 3’UTRs, although significant numbers of clusters also were observed in mRNA coding sequences (CDS) or in intronic regions (Fig 4C). Of the total number of 3’UTR miRNA binding clusters that were detected, 792 were computationally assigned to one of the EBV miR-BART miRNAs or to a miR-BART passenger strand, based on seed homology, including a moderate number of potential mRNA targets for the anti-apoptotic miRNAs miR-BART3, 6, 8, 16 and 22. Inspection of these mRNA targets revealed several with known pro-apoptotic activity including FEM1B and CASZ1a (miR-BART3), OCT1 (miR-BART6), ARID2 (miR-BART8), CREBBP and SH2B3 (miR-BART16) and finally PPP3R1, PAK2 and TP53INP1 (miR-BART22) (see S4 Table for a summary the known functions of these gene products and relevant citations). As noted above, all of these mRNAs contained 3’UTR targets, identified by PAR-CLIP, that bear full seed homology to the indicated miR-BART miRNA (Fig 5A). We therefore used PCR to clone the 3’UTRs of each of these human mRNAs (see S1 Table for sequence coordinates) and inserted these 3’ to the FLuc gene, as described in Fig 3. As may be observed (Fig 5B), every 3’ UTR tested conferred substantial inhibition of FLuc activity in cells co-expressing the cognate miR-BART miRNA that was statistically significant (p<0.05). All these 3’UTRs contained a single predicted miR-BART target site that was captured by PAR-CLIP except for CASZ1a, which also contained a captured site with seed homology to miR-BART18. However, this potential target was not responsive to co-expressed miR-BART18 in co-transfected cells (Fig 5B).

In order to test whether the suppression of Fluc activity by a given miR-BART is indeed due to the seed homology present in the clusters captured by PAR-CLIP, we introduced transversion mutations at the 3’UTR nucleotides pairing to miRNA seed positions 2, 4 and 6 in eight of the nine captured clusters in these 3’UTRs. In the ninth 3’UTR, derived from SH2B3, the predicted target site for miR-BART16 was removed by deletion. The 3’UTR of DICE1 was not mutated, as the single miR-BART3 binding site present in this 3’UTR has been previously validated (23). By assaying 3’UTR-containing FLuc reporters containing these mutations, in parallel with the wild-type 3’UTR-based FLuc reporters used in Fig 5B, we observed that loss of seed homology in the captured PAR-CLIP clusters resulted in the complete loss of miR-BART mediated inhibition of FLuc activity, consistent with our hypothesis that the clusters captured by PAR-CLIP are bound by the predicted miR-BARTs (S3A Fig). However, in the case of CASZ1a, mutation of the single predicted miR-BART3-5p target site only led to a partial recovery of FLuc activity, indicating an additional miR-BART3 target site(s) is present in the CASZ1a 3’UTR. Indeed, we computationally identified a potential site with seed homology to miR-BART3-3p (S3B Fig) that was not detected by PAR-CLIP but that could account for this residual inhibition. Although miR-BART3-3p is in principle a passenger strand, with miR-BART3-5p representing the dominant arm, both strands are actually recovered at similar levels and have a comparable number of binding clusters (Fig 4B). In conclusion, this mutational analysis confirmed that all 9 candidate 3’UTR target sites are indeed binding sites for the predicted miR-BART.

**Fig 6:**
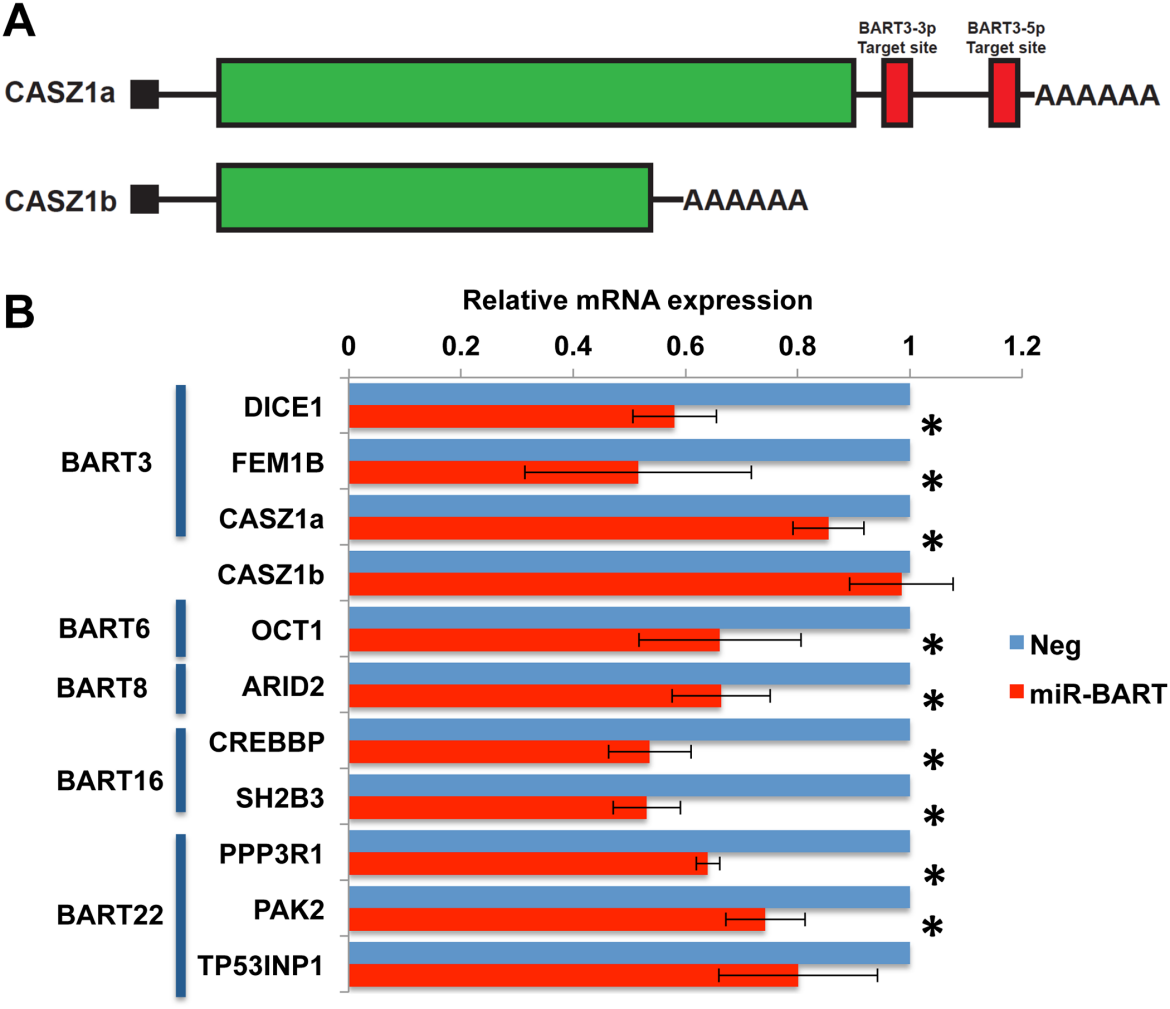
Expression of miR-BARTs in AGS cells reduced steady state mRNA levels for 10 candidate pro-apoptotic genes. (A) Schematic representation of two alternatively spliced isoforms of the CASZ1 mRNA, encoding CASZ1a and CASZ1b. Only the CASZ1a mRNA is predicted to contain miR-BART3 target sites. (B) Relative mRNA expression levels of candidate mRNAs in AGS cells transduced with lentiviral vectors expressing physiological levels of one of the 5 anti-apoptotic miR-BARTs (Fig 1), compared to control AGS cells (Neg; normalized to 1). Average of 3 experiments with SD indicated. *: Students’ T-Test, p<0.05.

If the pro-apoptotic genes listed in Fig 5 and S4 Table are indeed authentic targets of the miR-BART miRNA listed in the same figure, then expression of physiological levels of that miR-BART in AGS cells should result in a reduction in the expression of that gene [1]. We initially performed qRT-PCR analysis of control AGS cells and of the AGS cells described in Fig 1 that express close to physiological levels of one of the five anti-apoptotic miR-BARTs (Fig 2) looking at the nine cellular mRNAs listed in Fig 5 as well as the mRNA encoding DICE1, a previously reported [23] potentially pro-apoptotic target for miR-BART3 confirmed by indicator assay in Fig 3. As noted in Fig 6A, this analysis is more complex in the case of CASZ1, which is expressed in two spliced variants, encoding CASZ1a and a shorter protein called CASZ1b [39], as only the 3’UTR found in the longer mRNA splicing isoform encoding CASZ1a is predicted to be a target for miR-BART3.

As shown in Fig 6B, we observed a significant (p<0.05) reduction in the level of expression of all the predicted mRNA targets with the exception of the TP53INP1/miR-BART22 combination, where the observed reduction in mRNA expression fell slightly short of significance. Importantly, while expression of the CASZ1a mRNA was significantly reduced in the presence of pre-miR-BART3, expression of the CASZ1b mRNA was not, as predicted (Fig 6A).

**Fig 7:**
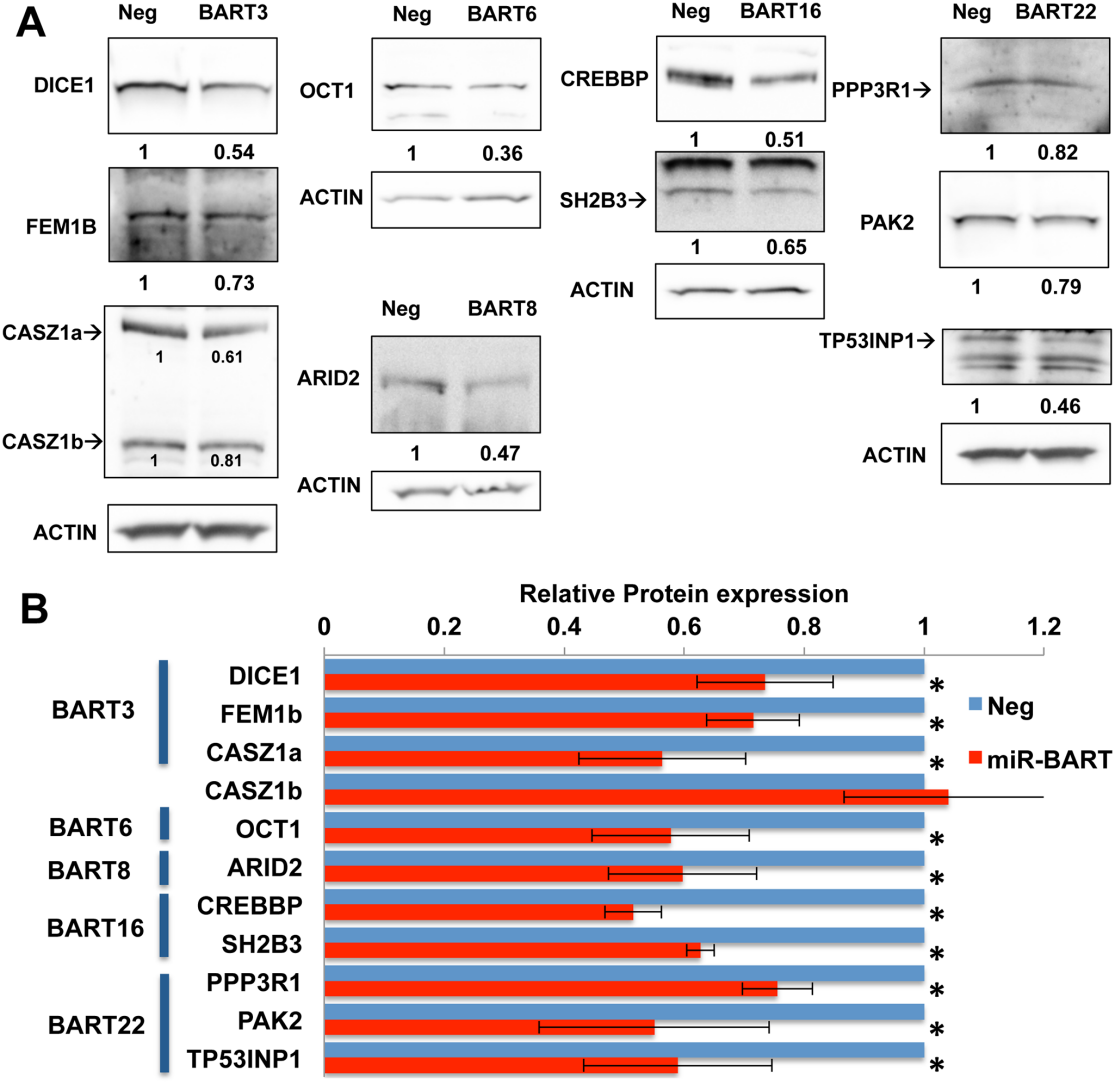
Expression of miR-BARTs in AGS cells suppressed protein expression from 10 candidate pro-apoptotic genes. (A) Representative Western blots showing the level of protein expression for 10 candidate target genes in AGS cells stably expressing the indicated miR-BART pre-miRNA compared to control AGS cells (Neg). Relative expression levels of each candidate gene product were normalized to an endogenous beta-actin control. Note that only CASZ1a expression was significantly inhibited by pre-miR-BART3, while CASZ1b expression was unaffected. Where more than one protein band was observed, the relevant band is indicated by an arrow. (B) Relative protein expression levels of candidate pro-apoptotic target genes in AGS cells stably expressing the indicated miR-BART pre-miRNA, compared to control AGS cells (Neg; normalized to 1). Average of four independent experiments with SD indicated. * Students’ T-Test, p<0.05.

While miRNAs can clearly reduce the steady-state expression level of target mRNAs, evidence suggests that a major, and possibly the primary, effect of RISC binding to the 3’UTR of a target mRNA is to reduce the translation of that mRNA [40, 41]. To address whether the anti-apoptotic miR-BARTs indeed reduce the expression of the proteins encoded by the 10 pro-apoptotic genes listed in Figs 5 and 6, we performed Western analyses of all ten proteins in the transduced AGS cells expressing the individual anti-apoptotic miR-BART miRNAs. Representative Western blots are shown in Fig 7A and a compilation of data derived from four independent experiments for each of the 10 proteins is shown in Fig 7B. As may be observed, these data uncover statistically significant (p<0.05) decreases in expression for all 10 cellular proteins under analysis, with the only non-repressed protein being CASZ1b which, as shown in Fig 6A, is encoded by a spliced mRNA isoform that is not expected to bind miR-BART3. In contrast, expression of the CASZ1a protein was, as expected, repressed upon miR-BART3 expression (Fig 7).

### Expression of artificial miRNAs specific for pro-apoptotic miR-BART target mRNAs phenocopies their anti-apoptotic effect

**Fig 8:**
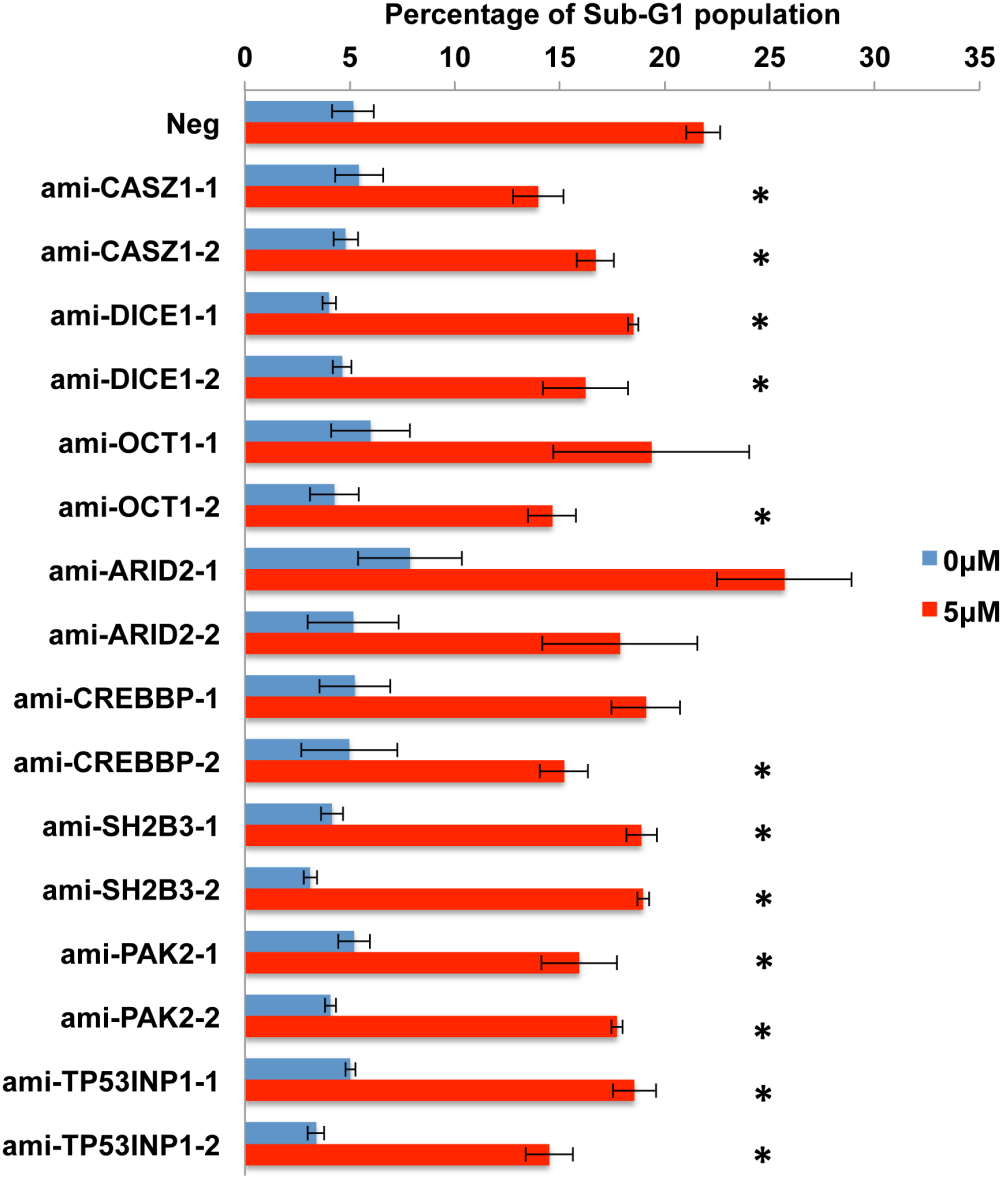
Expression of amiRNAs designed to repress candidate pro-apoptotic miR-BART target genes inhibits apoptosis in AGS cells. Each AGS culture was transduced with a control lentiviral vector (Neg) or with a lentiviral vector expressing an amiRNA specific for the indicated human gene and selected for puromycin resistance. The Sub-G1 population of AGS cells was determined by FACS analysis, in the absence or presence of 5 μM etoposide, at 24 h after drug addition and is given as a percentage of the total culture. Average of 3 independent experiments with SD indicated. *: Students’ T-Test, p<0.05.

The work described so far has identified several putatively pro-apoptotic cellular mRNA targets for the five anti-apoptotic EBV miR-BART miRNAs miR-BART3, 6, 8, 16 and 22 and shown that these are, in fact, downregulated at both the mRNA and protein level in AGS cells expressing physiological levels of the miR-BART miRNA in question. However, these data do not address whether this downregulation is, in fact, causatively related to the observed reduction in apoptosis. To test this hypothesis, we constructed two lentiviral vectors expressing artificial miRNAs (amiRNAs) specific for each of the 10 candidate mRNA targets, a total of 20 vectors [42]. These were used to transduce AGS cells that were then selected for blasticidin resistance and tested for knockdown of the encoded protein target by Western blot. As shown in S4 Fig, 16 distinct amiRNAs demonstrated some degree of knockdown ranging from >10-fold to as little as ~30%, for the targets CASZ1, OCT1, SH2B3, ARID2, PAK2, TP53INP1 and CREBBP as well as DICE1. Unfortunately, we did not observe significant knockdown of PPP3R1 or FEM1B with either amiRNA tested and these two potential targets were therefore not further addressed. However, we were able to test the other 16 amiRNA- expressing AGS cell lines for their ability to resist the induction of apoptosis seen upon incubation in 5 μM etoposide. As shown in Fig 8, we observed a statistically significant (p<0.05) reduction in apoptosis for both amiRNAs specific for CASZ1, DICE1, SH2B3, PAK2 and TP53INP1. We also observed a significant reduction in apoptosis in one of the two cell lines expressing an amiRNA specific for OCT1 and CREBBP, with the other cell line showing a trend towards lower apoptosis that did not achieve significance due to a high standard deviation between assay replicates. Finally, neither amiRNA specific for ARID2 resulted in reduced apoptosis, suggesting that this protein is perhaps not, in fact, functionally pro-apoptotic in AGS cells. In conclusion, our data demonstrate that amiRNAs specific for seven distinct cellular genes identified as targets for anti-apoptotic EBV miR-BART miRNAs are able to phenocopy the anti-apoptotic activity of these viral miRNAs.

## Discussion

The primary goal of this study was to determine if any of the miR-BART miRNAs expressed at high levels in EBV transformed epithelial cells have an anti-apoptotic phenotype and, if so, to identify and validate the cellular mRNA targets that mediate this phenotype. This work was initially prompted by published reports arguing that the expression of clusters of miR-BART miRNAs in the gastric carcinoma cell line AGS inhibits the apoptosis caused by exposure to the topoisomerase II inhibitor etoposide [20] and reports, based largely on computational approaches, that identified several individual pro-apoptotic cellular genes as potential targets for specific miR-BART miRNAs [14, 20–24].

Because the phenotypes exerted by miRNAs can be influenced by their expression level [28], we initially decided to construct stable cell lines, derived from human AGS cells, that individually expressed a close to physiological level of each of the miR-BART miRNAs using lentiviral vector transduction. As shown in Fig 1, we were indeed able to achieve a level of expression in AGS cells that was closely comparable to that seen in the naturally EBV transformed NPC cell line C666 for 17 of the 22 miR-BARTs. Analysis of these cell lines then showed that five of the EBV miRNAs, that is miR-BART3, 6, 8, 16 and 22, were able to reduce the level of apoptosis seen after etoposide treatment (Fig 2 and S1 Fig). We next globally identified the mRNA targets bound by RISC-loaded miR-BART miRNAs by PAR-CLIP analysis of the EBV-transformed epithelial cell line C666, using a pan-Ago antibody. This resulted in the identification of several cellular mRNA targets bound by the five anti-apoptotic miR-BARTs (Figs 4D and 5), nine of which were predicted to encode proteins with pro-apoptotic activity (S4 Table). We were able to further validate these cellular mRNAs as authentic targets for the five anti-apoptotic EBV miR-BART miRNAs by several criteria:

1. Insertion of the 3’ UTR, including the PAR-CLIP identified miR-BART seed target, 3’ to the FLuc indicator gene conferred specific downregulation of FLuc when the cognate miR-BART miRNA was expressed in *trans* (Fig 5B). Moreover, this downregulation was dependent on the integrity of the seed target (S3 Fig).
2. Expression of any one of these miR-BART miRNAs at physiological levels in AGS cells (Fig 1) resulted in a specific and significant downregulation of the level of expression of the endogenous mRNA and protein encoded by the predicted target gene (Figs 6 and 7).

While these four lines of evidence provide strong support for the hypothesis that the 10 genes listed in Figs 6 and 7 (nine of which are novel while one, DICE1, has been previously described [23]) are indeed authentic targets for one of the five anti-apoptotic miR-BARTs, they do not address whether these mRNA targets are directly relevant to the observed anti-apoptotic phenotype (Fig 2). To address this question, we constructed two artificial miRNA (amiRNA) lentiviral expression vectors specific for each of the potential miR-BART mRNA targets tested in Figs 6 and 7. These lentiviral vectors, which are closely similar to the miR-BART lentivectors used in Figs 1, 2, 6 and 7, were then used to generate stably transduced AGS cell lines expressing these amiRNAs. As shown in S4 Fig, we obtained two amiRNAs that each effectively and stably downregulated the expression of eight of these potentially pro-apoptotic genes in AGS cells (we did not obtain amiRNAs able to stably downregulate FEM1B or PPP3R1, either because our amiRNA designs were ineffective or because these proteins are required in AGS cells). Analysis of the resultant 16 stable knockdown AGS cell lines obtained showed a significant reduction in apoptosis levels after etoposide treatment in both cell lines expressing an amiRNA specific for CASZ1, DICE1, SH2B3, PAK2 or TP531NP1 and in one of the two cell lines expressing an amiRNA specific for OCT1 or CREBBP1. Neither amiRNA specific for ARID2 showed an anti-apoptotic phenotype, though both effectively inhibited ARID2 protein expression (S4D Fig). We therefore conclude that we have identified at least seven authentic pro-apoptotic cellular mRNA targets that are significantly downregulated upon expression of one of the anti-apoptotic miR-BART miRNAs at physiological levels in human epithelial cells. These findings can at least partly explain the previously reported anti-apoptotic activity of the miR-BART miRNA cluster in AGS cells [20].

Of the seven anti-apoptotic mRNA targets validated in this manuscript, i.e., CASZ1, DICE1, OCT1, CREBBP, SH2B3, PAK2 and TP53INP1, only one, DICE1 has been previously reported as an mRNA target for miR-BART3 [23]. This was surprising, as a number of other pro-apoptotic cellular mRNAs have also been reported to be targets for miR-BARTs [14, 20–22, 24, 33]. However, as shown in Fig 3 and S2 Fig, we were not able to validate STAT1, CASP3 or BIM as targets for any of the five pro-apoptotic EBV miRNAs miR-BART3, 6, 8, 16 and 22. It remains possible, as previously proposed [20], that the simultaneous expression of multiple miR-BARTs, as seen in EBV-transformed epithelial tumors, would result in significantly reduced expression of STAT1, CASP3 and/or BIM. However, we note that the 3’UTRs of STAT1 and CASP3, which have been reported to be targets for miR-BART8 and miR-BART16 respectively [14, 33], do not contain full seed targets for either of these two EBV miRNAs and neither 3’UTR was in fact identified as a target for miR-BART8 or miR-BART16 binding in the PAR-CLIP analysis reported in Fig 4D and S3 Table.

In addition to the previously reported mRNA targets for the anti-apoptotic miR-BARTs analyzed in Fig 3, several other potentially pro-apoptotic cellular mRNAs have also been previously reported as targets for other miR-BARTs that did not exert a detectable anti-apoptotic phenotype when expressed individually in AGS cells (Fig 2). These include BID, a proposed target for miR-BART4 [21]; PUMA, a proposed target for miR-BART5 [22]; PTEN, a proposed target for miR-BART7 [24]; E-Cadherin (E-CAD), a proposed target for miR-BART9 [43]; and finally, EBF1, a proposed target for miR-BART11 [44]. All of these miRNAs, except miR-BART4, were expressed at physiological levels in the AGS transductants (Fig 1), so the lack of a detectable anti-apoptotic phenotype was unexpected.

Analysis of our PAR-CLIP data, obtained in C666 cells, as well as previous PAR-CLIP experiments, using Ago-specific antibodies, performed using LCLs or PEL cells latently infected with wildtype EBV [11, 37], and expressing readily detectable levels of the miR-BARTs, failed to identify miR-BART binding sites at the proposed locations in the 3’UTRs of any of these mRNAs (S3 Table). Moreover, FLuc-based indicator constructs containing 3’UTRs derived from these five mRNA species either failed to show any evidence of downregulation in the presence of the cognate miR-BART expression plasmid (BID/miR-BART4; PTEN/miR-BART7; E-CAD/miR-BART9) or showed a minimal level of inhibition (PUMA/miR-BART5 and EBF1/BART11) (S5 Fig). We note that the EBF1 3’UTR does not, in fact, contain a seed target for miR-BART11 and is therefore not predicted to be highly responsive to this miRNA. Others have also failed to confirm the identification of PUMA as an authentic target for miR-BART5 using RISC immunoprecipitation or indicator assays [14, 20], so the PUMA 3’UTR, despite the presence of a highly complementary potential 3’UTR target, may not in fact be an authentic target for downregulation by miR-BART5.

In conclusion, we have identified a series of at least seven mRNA targets for EBV miR-BART miRNAs that encode pro-apoptotic proteins. The BART miRNA-induced reduction in the expression of these proteins can at least partly explain the previously reported anti-apoptotic activity of the EBV miR-BART locus in EBV latency II epithelial cells [20]. Clearly, this activity could be highly advantageous to EBV in ensuring the survival of these latently infected cells despite the known ability of EBV to activate innate immune pathways that have the potential to induce programmed cell death pathways [45] and may also contribute to the development of resistance seen in a significant percentage of EBV+ NPC tumors in patients undergoing chemotherapy or radiation therapy [46, 47].

## Materials and Methods

### Cell cultures and plasmids

C666 cells (a gift from Dr. Nancy Raab-Traub) [7], AGS cells (a gift from Dr. Lindsey Hutt-Fletcher) [16] and 293T cells (Duke Cancer Institute Cell Culture Facility) were cultured using RPMI 1640, Ham’s F-12 and Dulbecco's Modified Eagle Medium (Gibco), respectively, supplemented with 10% fetal bovine serum and 10 μg/ml gentamicin. All cell cultures were maintained at 37C with 5% CO_2_.

Lentiviral miR-BART miRNA expression vectors used for FLuc-based 3’UTR reporter assays were generated in the pLenti-CMV-Blasticidin (pLCB) backbone, and individual ~300 bp EBV miRNA expression regions, as previously described [30], were inserted into the 3’UTR of the Blasticidin gene using unique XhoI and XbaI sites. Functional expression of individual miR-BART miRNAs was confirmed using miRNA indicator assays [30] and stem-loop-qRT-PCR (Fig 1).

Lentiviral miR-BART miRNA expression vectors used for transduction of AGS cells were generated in the pTRIPZ backbone (Open Bioystems) (doxycycline inducible turboRFP, puromycin selectable), with EBV miRNA expression regions inserted into the 3’ UTR of the turboRFP gene using XhoI and EcoRI sites.

FLuc-based 3’UTR reporter plasmids were generated using the pLenti-SV40-GL3 (pLSG) backbone [37] by inserting 3’UTRs of candidate cellular target mRNAs (see S1 Table for full description of the inserted sequences) into the 3’UTR of FLuc between unique XhoI and XbaI sites. PCR primers used to clone the 3’UTRs are listed in S1 Table. To generate mutant 3’UTR reporter plasmids, internal primers bearing transversion mutations of the nucleotides pairing to seed positions 2, 4 and 6 of the miRNA were utilized, together with the primers listed in S1 Table, to clone mutant forms of the 3’UTR regions from the wild-type 3’UTR reporter plasmids by overlap extension PCR. To clone a truncated SH2B3 3’UTR, an internal forward primer and the reverse primer listed in S1 Table were utilized. The PCR primers used to clone the mutant 3’UTRs are listed in S7 Table.

Lentiviral amiRNA expression vectors were generated in the pLenti-CMV-Blasticidin-Hairpin (pLCBH) vector. pLCBH was derived from pLCB by inserting a miR-30-based amiRNA cassette [42] into the 3’UTR of the blasticidin gene between the unique XhoI and EcoRI sites. Oligonucleotides used to clone specific amiRNAs are listed in S6 Table.

### qRT-PCR and stem-loop qRT-PCR

qRT-PCR for determination of relative mRNA expression and stem-loop qRT-PCR for relative miRNA expression were performed based on vendor protocols. Briefly, total RNA was first harvested using TRIzol (Ambion). For qRT-PCR analysis, RNAs were reverse transcribed using a high capacity reverse transcription kit (Applied Biosystems) and assayed with Power SYBR Green PCR Master Mix (Applied Biosystems). Relative gene expression was first normalized to GAPDH and was then compared to the negative control. Primers used to detect distinct isoforms of CASZ1 mRNA were as previously described [39]. All the qPCR primers used are listed in S5 Table.

For stem-loop qRT-PCR, total RNA preparations were reverse transcribed using a Taqman miRNA reverse transcription kit (Applied Biosystems), and assayed with Taqman Universal PCR Master Mix, no UNG (Applied Biosystems). The relative miRNA expression level of individual miR-BART miRNAs expressed in transduced AGS cells was first normalized to endogenous U6, and then to the miR-BART miRNA level detected in C666 cells. All the EBV miR-BART reverse transcription primers and stem-loop qPCR probes were purchased from Life Technologies.

### Sub-G1 apoptosis assay

10^5^ AGS cells were plated into each well of a 6-well plate, and after 24 h, a final concentration of 5 μM/ml etoposide (SigmaAldrich) (25 μM/ml for PARP cleavage) was added to the medium. After 24 h, both floating and adherent cells were harvested, pooled together and fixed with 80% ethanol for ~4 h. Cells were then stained with PI solution with RNase A (BD Pharmingen) and analyzed by flow cytometry. Data were further analyzed by FlowJo (Treestar).

### Western blot analysis

Cells were lysed in NP40 lysis buffer supplemented with Complete Mini EDTA-free proteinase inhibitors (Roche). Cell lysates were separated by SDS-PAGE and subsequently transferred to nitrocellulose membranes. Western blots were probed using primary antibodies including anti-FEM1B (sc-67568, Santa Cruz), anti-CASZ1 (sc-135453, Santa-Cruz), anti-DICE1 (sc-376524, Santa Cruz), anti-OCT1 (sc-232, Santa Cruz), anti-ARID2 (sc-166117, Santa Cruz), anti-CREBBP (sc-369, Santa Cruz), anti-SH2B3 (sc-393709, Santa Cruz), anti-PAK2 (sc-1872, Santa Cruz), anti-PPP3R1 (sc-6119, Santa Cruz), anti-TP53INP1 (sc-689919, Santa Cruz), anti-beta-Actin (sc-47778, Santa Cruz), and anti-PARP (9542p, Cell Signaling). The secondary antibodies used included anti-Goat IgG (sc-2020, Santa Cruz), anti-Mouse IgG (A9044, Sigma) and anti-Rabbit IgG (A6145, Sigma). All images were obtained using G:BOX (Syngene) and GeneSys (Syngene) acquisition software, and were subsequently analyzed by Genetools software (Syngene).

### Transfections and luciferase 3’UTR reporter assays

10 ng of a pLSG-based 3’UTR reporter, 10 ng pLenti-SV40-Rluc, along with either 500 ng of a miR-BART expression vector or a matched negative control, were co-transfected into 293T cells in 24-well plates using polyethylenimine (PEI). Cells were lysed ~72 h post-transfection with passive lysis buffer (Promega) and FLuc and RLuc expression analyzed using a dual luciferase assay kit (Promega). All 3’UTR reporter assays were performed on three separate occasions using technical triplicates.

### Generation of the small RNA deep sequencing library

The small RNA deep sequencing library for C666 cells was generated as previously described [11]. Briefly, C666 total RNA was first harvested using TRIzol (Ambion), and the small RNA fraction (~18 to ~24 nt) was subsequently isolated using 15% TBE-Urea polyacrylamide gels (Bio-Rad). The harvested RNAs were then ligated to 3’ and 5’ Illumina adapters, reverse transcribed using SSIII (Invitrogen) and subjected to Illumina deep sequencing.

### Generation of the PAR-CLIP deep sequencing library

The PAR-CLIP library for C666 was generated as previously described [11, 37]. Briefly, C666 cells were first expanded to 30 150-mm dishes at ~80% confluency, and were then cultured in the presence of 100 μM 4-thiouridine (4SU) for ~20 h. The cells were then UV radiated at 365 nm for 1 minute, harvested and lysed on ice in NP40 lysis buffer. Cross-linked Ago:RNA complexes were then immunoprecipitated using a pan-Ago antibody (ab57113; Abcam) and protein G Dynabeads (Invitrogen). Ago-bound RNAs were digested with RNaseT1, radio-labeled, gel purified, proteinase K treated, phenol-chloroform extracted, ethanol precipitated and ligated to 3’ and 5’ Illumina adapters. After reverse transcription and limited PCR amplification, the recovered cDNAs were deep-sequenced.

### Bioinformatics

The C666-derived small RNA deep sequencing library and PAR-CLIP library were processed as previously described [11, 37]. Briefly, sequencing reads were pre-processed using the FAST-X toolkit (http://hannonlab.cshl.edu/fastx_toolkit/), and were aligned to the human genome (hg19) and EBV1 wild type genome using Bowtie with up to two (three for PAR-CLIP) mismatches allowed.

The PAR-CLIP library was further processed using the PARalyzer program, as previously described [11, 37, 38]. Briefly, reads were first filtered allowing for up to three mismatches but with only one or zero non-T-to-C mutations. Subsequently, reads that aligned to a unique genomic location, that contained at least one T-to-C mutation and that overlapped by at least one nucleotide were grouped together as clusters. Clusters with a read depth of at least five reads were presented as miRNA:mRNA interaction sites in the PAR-CLIP dataset. Each cluster in the PAR-CLIP dataset was further examined for canonical miRNA seed match sites, using the miRNA expression data generated from the small RNA deep sequencing library derived in parallel, and miRNAs with seed matches equal to or greater than 7mer1A (perfect base pairing to seed nt 2–7 with an A across from nt 1 of the miRNA, see [5]) to the cluster were identified as candidate miRNAs putatively responsible for the cluster. The raw sequencing data from the C666 small RNA deep sequencing and PAR-CLIP analysis have been submitted to the NCBI Sequence Read Archive (SRA), and both dataset can be accessed with the accession number GSE67990. The sub-accession numbers of the individual C666 small RNA deep sequencing and PAR-CLIP libraries are GSM1660655 and GSM1660656, respectively.

## Supporting Information

S1 Fig Several miR-BART miRNAs reduce the level of PARP cleavage induced by etoposide treatment of AGS cells. (A) Western blot showing PARP cleavage in AGS cells transduced with a negative control lentivector, or with a vector expressing an anti-apoptotic miR-BART, in response to no drug treatment or incubation in 25 μM etoposide for 24 h. The upper band corresponds to full-length PARP while the lower band represents cleaved PARP. A Western blot of beta-actin is also shown as a loading control. (B) The ratio of cleaved to uncleaved PARP detected after incubation with 25 μM etoposide. Average of 3 experiments with SD indicated. The cleaved to uncleaved PARP ratios seen in the negative control cultures in each experiment were set at 1.
(TIF)

S2 Fig 3’UTR luciferase reporter assays of BIM and CASP3. This assay was performed as described in Fig 3 using FLuc-based indicator plasmids containing the BIM or CASP3 3’UTR. (A) Relative BIM 3’UTR reporter activity in 293T cells co-transfected with vectors expressing the indicated miR-BART, compared to the negative control. (B) Relative CASP3 3’UTR reporter expression in 293T cells co-transfected with vectors expressing the indicated miR-BART, compared to the negative control. Average of 3 independent experiments with SD indicated.
(TIF)

S3 Fig Luciferase reporter assays using wild-type and mutant 3’UTRs. This assay was performed as described in Fig 3 using Fluc-based indicator plasmids containing wild-type or mutant candidate gene 3’UTRs. (A) Relative wild-type (in blue bars) and mutant 3’UTR (in green bars) FLuc activity detected in 293T cells co-transfected with vectors expressing the indicated miR-BART, compared to a negative control vector (Neg). Average of 3 independent experiments with SD indicated. (B) Sequence alignment of a computationally identified target site for miR-BART3-3p present in the CASZ1a 3’UTR.
(TIF)

S4 Fig Stable expression of amiRNAs targeted to candidate pro-apoptotic genes represses expression of the encoded protein. (A) to (H) Stable expression in AGS cells of one of two distinct amiRNAs designed to target mRNA transcripts derived from each candidate pro-apoptotic gene, using lentiviral vectors, results in reduced expression of the encoded protein. Relative expression levels, shown below each lane, were normalized to an endogenous beta-actin control and to the expression level seen in negative control (Neg) AGS cells, which was set at 1.
(TIF)

S5 Fig Stable expression of physiological levels of miR-BART miRNAs fails to effectively repress several previously published mRNA 3’UTR targets. Upper panel: FLuc-based reporter plasmids containing the 3’UTR derived from the indicated cellular mRNAs were co-transfected into 293T cells along with a vector expressing the indicated pre-miR-BART, or an empty vector, as well as an RLuc-based internal control plasmid. At 72 h post-transfection, induced Fluc and RLuc activities were determined and normalized to RLuc. The negative control was set at 1. Average of three independent experiments with SD indicated. Lower panel: published mRNA targets of miR-BARTs without significant anti-apoptotic potential in AGS cells (Fig 2), with their predicted pro-apoptotic activity indicated by + or +/-, if this is uncertain. None of the cellular 3’UTRs shown here contains a miR-BART-dependent RISC binding site identified by PAR-CLIP, as performed here using C666 cells, or previously using LCLs or primary effusion lymphomas (PEL) cells infected with WT EBV [11, 37]. The red line indicates the minimum predicted response of an authentic 3’UTR target in this assay format.
(TIF)

S1 Table Derivation of FLuc-based reporter plasmids based on cellular mRNA 3’UTRs. Shown are the sequences of the two PCR primers used to clone human mRNA 3’UTRs, using mRNA obtained from 293T cells, the native 3’UTR length, the PCR-amplified 3’UTR length used in the indicator plasmids, the location of the BART miRNA binding clusters identified by the PAR-CLIP performed here using C666 cells and the miR-BART interaction sites on mRNA 3’UTRs proposed by others and reported in the literature.
(XLSX)

S2 Table Small RNA deep sequencing library from C666 cells. Shown are mature miRNA or passenger strand species expressed in C666 cells and captured by small RNA deep sequencing. Information including miRNA names, expression loci on the human or EBV genome, number of reads, percentage expression of miRNAs from pre-miRNA precursors, actual captured sequences and pre-miRNA sequences are provided.
(XLS)

S3 Table PAR-CLIP library obtained from C666 cells using a pan-Ago antibody. Shown are RISC-associated clusters captured by PAR-CLIP in C666 cells after Ago immunoprecipitation. Information including binding cluster loci on the human or EBV genome, computationally predicted miRNAs targeting these clusters, read counts, and cluster locations on mRNAs (3’UTR, 5’UTR, intron or intergenic regions) are provided.
(XLSX)

S4 Table Current literature on 10 pro-apoptotic candidate genes. Listed is the published evidence arguing that these 10 candidate target mRNAs for the anti-apoptotic miR-BARTs encode cellular proteins that are pro-apoptotic and/or anti-proliferative. Information including key findings, references and PMID are shown.
(XLSX)

S5 Table Primers used to perform the qRT-PCR assays reported in this manuscript. (XLSX)

S6 Table Oligonucleotides used to construct amiRNA expression vectors. (XLSX)

S7 Table PCR primers used to generate mutant 3’UTRs. (XLSX)
