# Supplementary figures and images for: EBV BART MicroRNAs Target Multiple Pro-apoptotic Cellular Genes to Promote Epithelial Cell Survival
Source: PLoS Pathog. 2015 Jun 12;11(6):e1004979. doi: 10.1371/journal.ppat.1004979 (PMC4466530; doi:10.1371/journal.ppat.1004979)

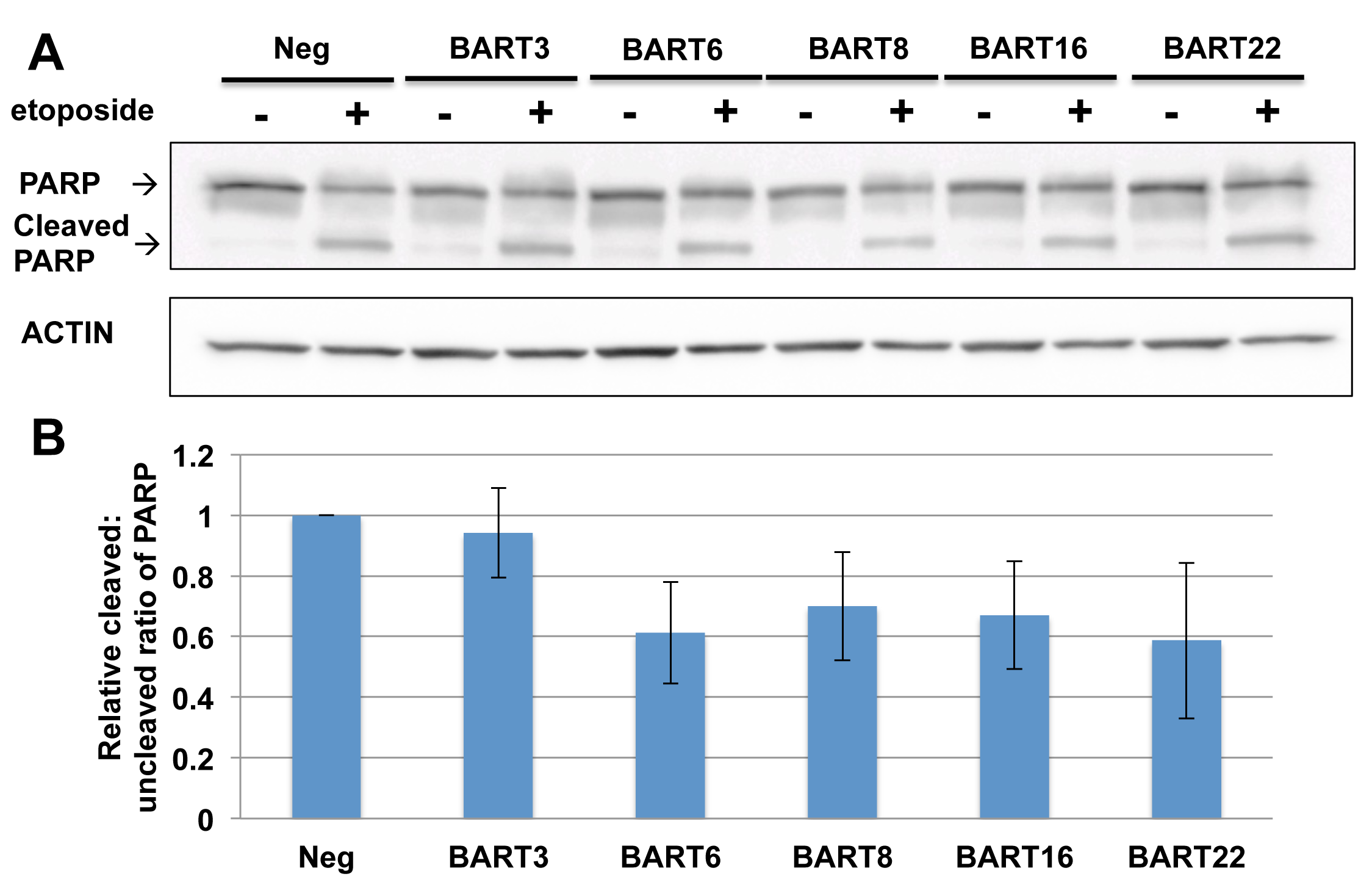

Supplement: S1 Fig — (A) Western blot showing PARP cleavage in AGS cells transduced with a negative control lentivector, or with a vector expressing an anti-apoptotic miR-BART, in response to no drug treatment or incubation in 25 μM etoposide for 24 h. The upper band corresponds to full-length PARP while the lower band represents cleaved PARP. A Western blot of beta-actin is also shown as a loading control. (B) The ratio of cleaved to uncleaved PARP detected after incubation with 25 μM etoposide. Average of 3 experiments with SD indicated. The cleaved to uncleaved PARP ratios seen in the negative control cultures in each experiment were set at 1. (TIF) [file ppat.1004979.s001.tif]

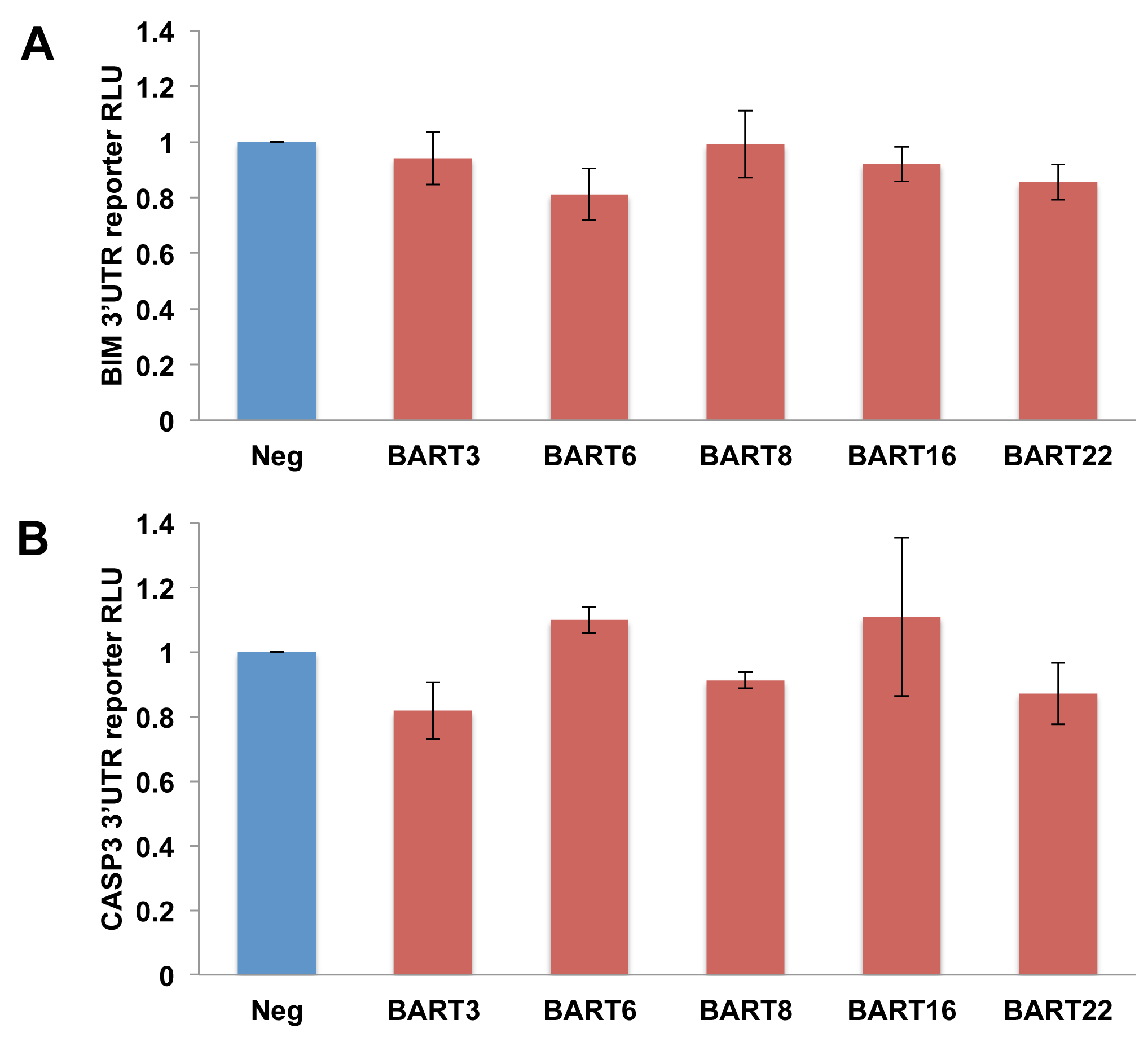

Supplement: S2 Fig — This assay was performed as described in Fig 3 using FLuc-based indicator plasmids containing the BIM or CASP3 3’UTR. (A) Relative BIM 3’UTR reporter activity in 293T cells co-transfected with vectors expressing the indicated miR-BART, compared to the negative control. (B) Relative CASP3 3’UTR reporter expression in 293T cells co-transfected with vectors expressing the indicated miR-BART, compared to the negative control. Average of 3 independent experiments with SD indicated. (TIF) [file ppat.1004979.s002.tif]

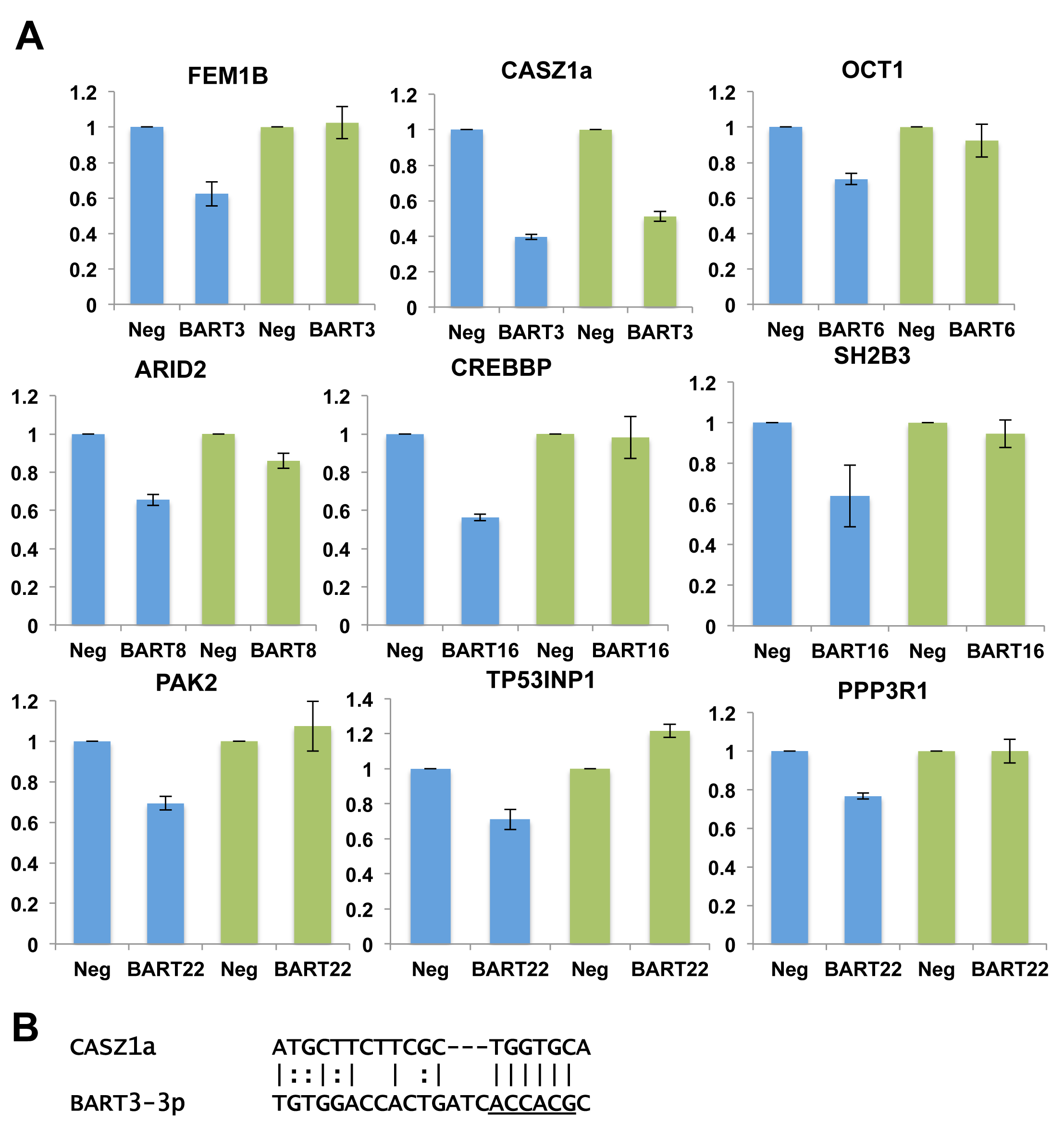

Supplement: S3 Fig — This assay was performed as described in Fig 3 using Fluc-based indicator plasmids containing wild-type or mutant candidate gene 3’UTRs. (A) Relative wild-type (in blue bars) and mutant 3’UTR (in green bars) FLuc activity detected in 293T cells co-transfected with vectors expressing the indicated miR-BART, compared to a negative control vector (Neg). Average of 3 independent experiments with SD indicated. (B) Sequence alignment of a computationally identified target site for miR-BART3-3p present in the CASZ1a 3’UTR. (TIF) [file ppat.1004979.s003.tif]

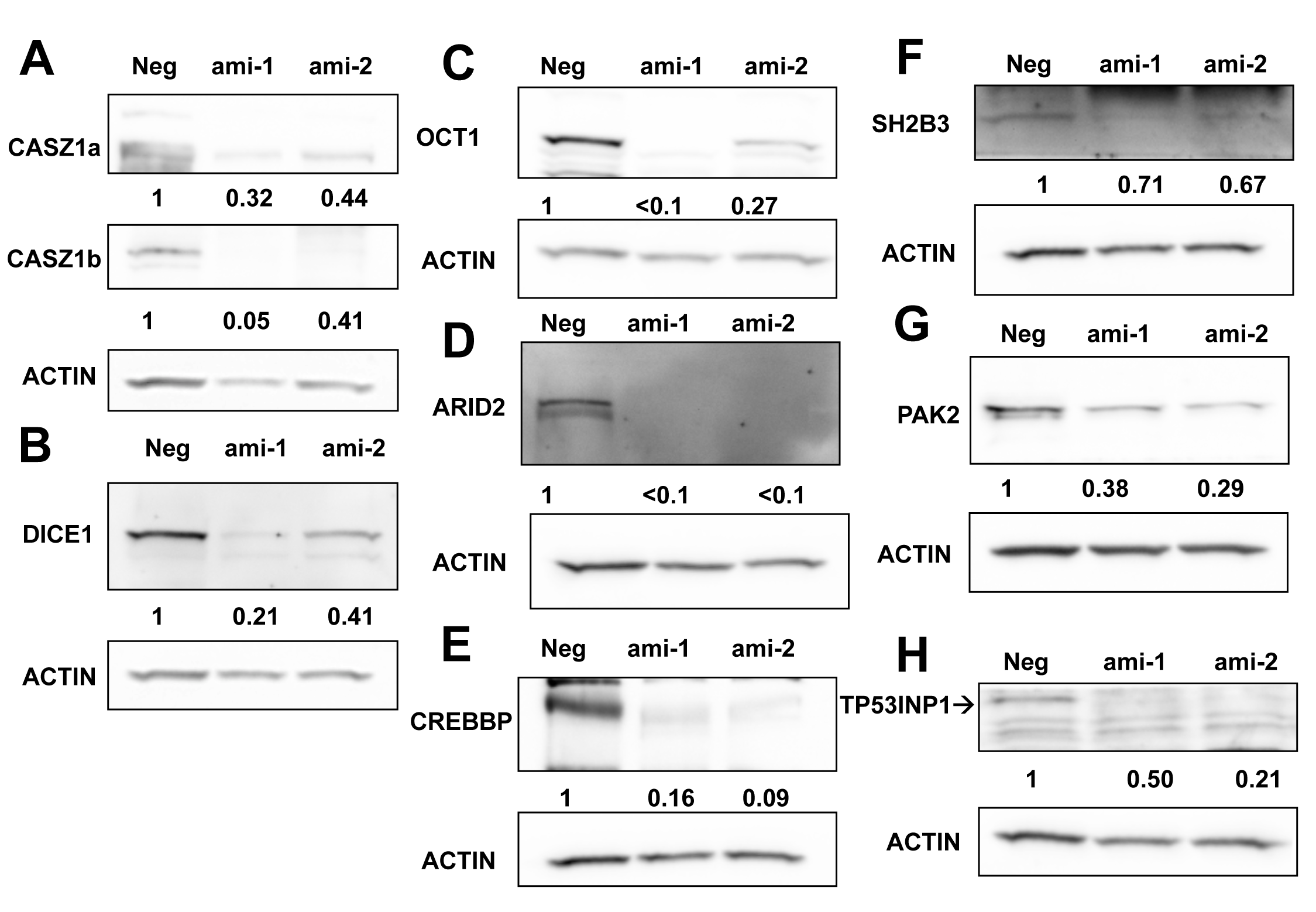

Supplement: S4 Fig — (A) to (H) Stable expression in AGS cells of one of two distinct amiRNAs designed to target mRNA transcripts derived from each candidate pro-apoptotic gene, using lentiviral vectors, results in reduced expression of the encoded protein. Relative expression levels, shown below each lane, were normalized to an endogenous beta-actin control and to the expression level seen in negative control (Neg) AGS cells, which was set at 1. (TIF) [file ppat.1004979.s004.tif]

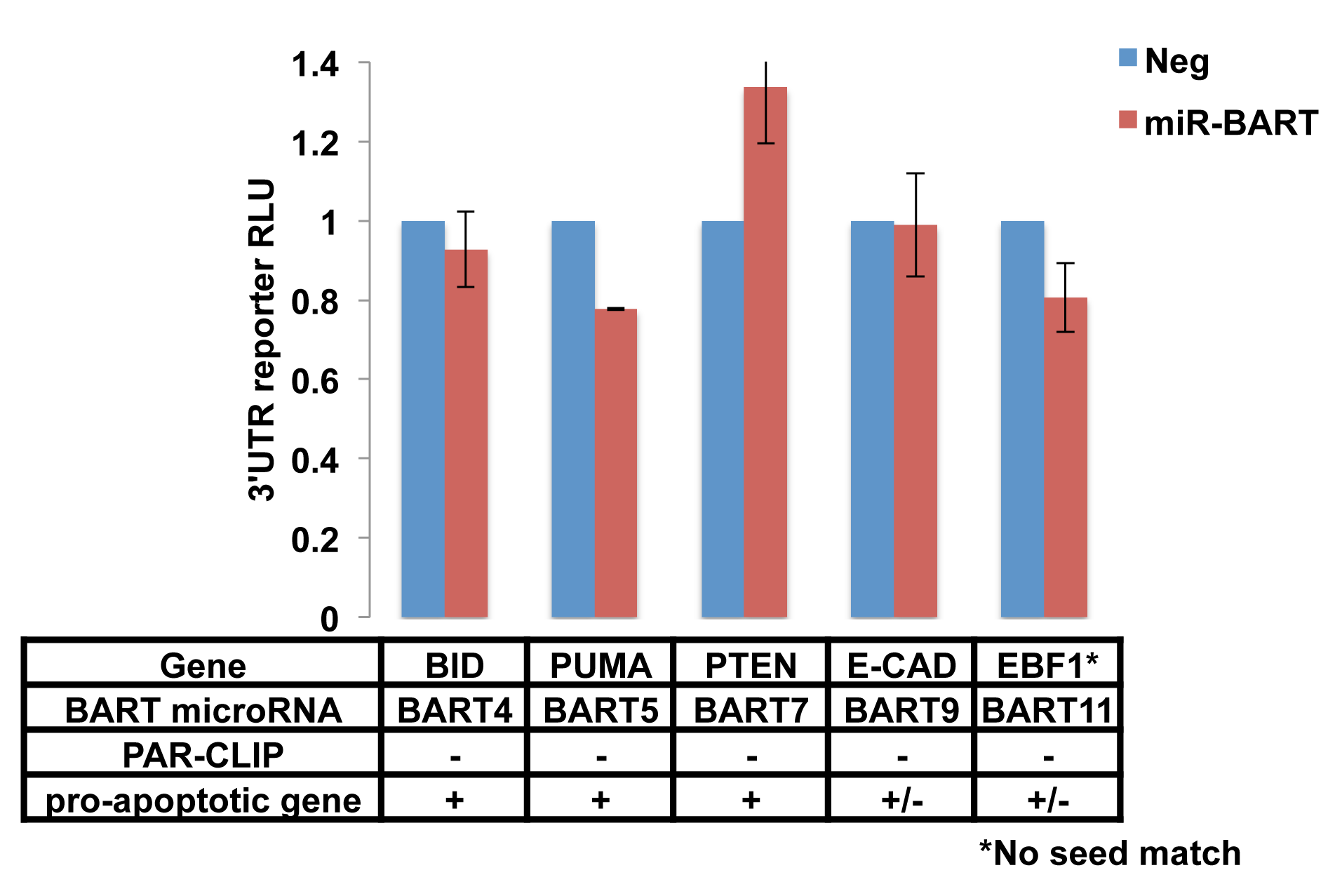

Supplement: S5 Fig — Upper panel: FLuc-based reporter plasmids containing the 3’UTR derived from the indicated cellular mRNAs were co-transfected into 293T cells along with a vector expressing the indicated pre-miR-BART, or an empty vector, as well as an RLuc-based internal control plasmid. At 72 h post-transfection, induced Fluc and RLuc activities were determined and normalized to RLuc. The negative control was set at 1. Average of three independent experiments with SD indicated. Lower panel: published mRNA targets of miR-BARTs without significant anti-apoptotic potential in AGS cells (Fig 2), with their predicted pro-apoptotic activity indicated by + or +/-, if this is uncertain. None of the cellular 3’UTRs shown here contains a miR-BART-dependent RISC binding site identified by PAR-CLIP, as performed here using C666 cells, or previously using LCLs or primary effusion lymphomas (PEL) cells infected with WT EBV [11, 37]. The red line indicates the minimum predicted response of an authentic 3’UTR target in this assay format. (TIF) [file ppat.1004979.s005.tif]
